# Methotrexate carried in lipid core nanoparticles reduces microglial activation and induces neuroprotection after cortical stroke induced in rats

**DOI:** 10.1016/j.clinsp.2025.100676

**Published:** 2025-05-11

**Authors:** Edmundo L.R. Pereira, Raul C. Maranhão, Michelle N.C. Dias, Ijair R. dos Santos, Carolina Ramos dos Santos, Moisés Hamoy, Danielle Cristine A. Feio, Priscila O. Carvalho, Jaqueline M. Bazioli, Aleksandra T. Morikawa, Walace Gomes-Leal

**Affiliations:** a Laboratory of Experimental Neuroprotection and Neuroregeneration, Institute of Collective Health, Universidade Federal do Oeste do Pará, Santarém, PA, Brazil; b Neurology Unity, Hospital Universitário João de Barros Barreto, Universidade Federal do Pará, Belém, PA, Brazil; c Laboratório de Metabolismo e Lípides, Instituto do Coração (InCor-HCMUSP), Department of Cardiopneumology of the Faculdade de Medicina da Universidade de São Paulo, São Paulo, SP, Brazil; d Laboratory of Toxicology and Natural Products, Institute of Biological Sciences, Universidade Federal do Pará, Belém, PA, Brazil; e Laboratory of Molecular Biology, Universidade Estadual do Pará, Belém, PA, Brazil

**Keywords:** Drug delivery, Neuroinflammation, Neuroprotection, Methotrexate (Methotrexate), Solid lipid nanoparticles

## Abstract

*Background* This study aimed to investigate the effects of LDE-MTX on acute cerebral infarction. The study focuses on how LDE-MTX can influence the outcomes of ischemic stroke in a rat model, specifically examining its neuroprotective properties.

*Methods* Radioactively labeled LDE uptake by brain tissue was determined after IV injection in rats with Endothelin-1 (ET-1)-induced cortical ischemic stroke (*n* = 11) and controls (*n* = 18). Two groups of 5 animals were treated with LDE-MTX (1 mg/kg, IV) or LDE-alone 4 h post-stroke induction. After 7days, tissues were analyzed by immunohistochemistry for neuronal bodies, astrocytes, and microglia.

*Results* LDE uptake was fivefold higher in ischemic rats than in controls (*p* = 0.0003). LDE-MTX significantly inhibited microglial activation, resulting in a tenfold decrease in activated macrophages, and increased neuronal survival by 319 % in the periinfarct area. LDE-MTX had no effect on astrocytosis or primary infarct size.

*Conclusions* LDE-MTX demonstrated neuroprotective effects and shows potential as a novel strategy to limit ischemic stroke damage. The results suggest that LDE-MTX could be a promising treatment option for reducing ischemic damage in stroke patients, particularly through its effect on microglial activation and neuronal survival.

## Highlights

• MTX in lipid core nanoparticles (LDE) shows low toxicity and reduces inflammation.
• LDE-MTX treatment reduces microglial activation and macrophage count in rats.
• LDE-MTX boosts neuroprotection, increasing neuronal survival by 319 %.
• LDE-MTX reduces ischemic stroke damage in rat models.
• Potential novel treatment for ischemic stroke patients.

## Introduction

Ischemic brain stroke is one of the major manifestations of atherosclerosis, together with acute myocardial infarction, and represents one of the most frequent causes of death and disability in the world.^1^^,^^2^ In this condition, following the paucity of glucose and oxygen in the local tissue, a core infarcted area is surrounded by an area of ischemic penumbra, in which the neural dysfunction is still reversible. However, the necrotic core tends to expand over the ischemic penumbra.^3^^,^^4^ In this setting, it is of chief importance to develop neuroprotective agents to avoid further damage to the brain and contribute to the functional recovery of the ischemic penumbra. Drugs that promote inhibition of the excitatory amino acid pathways have been shown to be protective of the neurons and the glia in experimental animals.^5^ However, in several clinical studies enrolling patients with brain stroke, no consistent beneficial effects of those drugs have been proven.

Maranhão et al.^6^ developed a drug-targeting system consisting of artificially made nanoparticles, termed LDE that resembles the lipid composition of Low-Density Lipoprotein (LDL) but without the LDL protein moiety, apolipoprotein (apo) B. When LDE nanoparticles are in contact with the plasma, they acquire apo E. Apo E is recognized by the LDL receptors on the cell plasma membrane, which allows LDE to be taken up by the cells similarly to native LDL.^6^ Hence, drugs carried in LDE are delivered to the cytoplasm via the LDL receptor-mediated endocytic pathway.

LDE has been used as an efficient carrier for the transport of chemotherapeutic agents such as paclitaxel,^7^, ^8^, ^9^ Methotrexate (MTX),^9^, ^10^, ^11^, ^12^, ^13^, ^14^ carmustine,^15^, etoposide^16^ and docetaxel,^17^, in different experimental models of diseases and in clinical trials for treatment of solid and non-solid tumors^18^^,^^19^ and for treatment of patients with aortic atherosclerotic disease,^20^ coronary artery disease and endometriosis. In all tested LDE-drug formulations, the pharmacological action of the carried drugs was higher compared to the commercial conventional presentations,^18^ and the toxicity was drastically decreased when those drugs were associated with LDE.

MTX is widely used as an anti-inflammatory agent mainly in rheumatic diseases and it was found that MTX associated with LDE increased the uptake of the drug by cells.^21^ Subsequently, it was shown that LDE-MTX has the ability to markedly reduce the area of atherosclerotic lesions of cholesterol-fed rabbits and to decrease the invasion of the arterial intima by macrophages and smooth cells.^10^

LDE-MTX treatment was also effective in reducing the infarction size and improving the cardiac function in rats with acute myocardial infarction induced by ligation of the left coronary artery.^14^ In contrast, the effects of commercial, free MTX were minor. Taken together, the results from those studies prompted us to test the hypothesis of whether treatment with LDE-MTX could also have beneficial effects on a rat model of ischemic acute encephalic infarction.

## Material and methods

### Ethical and legal aspects

Male adult Wistar rats (250‒300 g) were obtained from the Central Animal Facility of the Federal University of Pará. All animals were housed under standard conditions with food and water available ad libitum. This study was conducted following the ARRIVE guidelines. All experimental procedures were carried out in accordance with the Principles of Laboratory Animal Care (NIH publication n° 86-23, revised 1985) and European Commission Directive 86/609/EEC for animal experiments under the license of the Ethics Committee on Experimental Animals of the Federal University of Para (approval number BIO008-11).

### Study design

#### Experimental model of cortical ischemia

Focal cortical ischemia was induced by microinjections of Endothelin-1 (ET-1), a potent vasoconstrictor that in brain microinjections causes a considerable reduction of blood flow.^22^ The ET-1 model of stroke was previously validated in several studies.^23^^,^^24^

In short, animals were anesthetized with ketamine hydrochloride (72 mg/kg, i.p) and xylazine hydrochloride (9 mg/kg, i.p) and held in a stereotaxic frame (Insight, Ribeirão Preto, Brazil) after their corneal and paw withdraw reflexes were abolished. A homoeothermic blanket unit was used to maintain the body temperature of the animals, measured by a rectal thermometer. After craniotomy, 40 pmoL of ET-1 (Sigma-Aldrich, Saint Louis, MO, USA) in 1 μL of sterile saline was injected into the rat motor cortex over a period of 2 min. using a finely drawn glass capillary needle. The capillary needle was left in position for 3 min. before being slowly withdrawn. Control animals were injected with the same volume of sterile saline. The authors used the following stereotaxic coordinates in relation to the bregma: +2.3 mm lateral; +1.2 mm posterior and 0.50 mm deep from the pial surface in the dorsoventral axis.^25^ To identify the injection site, a small quantity of colanyl blue was added to both ET-1 and vehicle solutions. After surgery, animals were allowed to recover with free access to food and water for 7-days.

#### Animal groups and treatments

Two experimental protocols were designed for the present study.

Protocol 1 was designed to evaluate the LDE uptake by the brain; animals were allocated to two groups a Control group of 18 animals without ischemia and an Ischemic group of 11 animals. Both groups were injected via the caudal vein with 200 μL (480,000 cpm) LDE labeled with 3H-cholesteryl oleate ether (3H-LDE). Twelve hours after injection of the labeled LDE the animals were euthanized, and tissues and organs were excised for radioactive uptake quantification.

Protocol 2 was designed to test the pharmacological potential of LDE-MTX. The experimental animals were allocated to two groups:

- LDE-MTX group: five animals with induced cerebral ischemia were treated with LDE-MTX (1 mg/kg MTX) injected through the caudal vein 4 hours after ischemia induction.
- LDE-alone group: five animals with induced cerebral ischemia were injected with LDE only through the caudal vein 4 h after ischemia induction.

The animals of both groups were euthanized for analysis 7 days after treatments.

### LDE preparation and association with MTX

LDE was prepared at the Laboratory of the Metabolism and Lipids of the Heart Institute, University of São Paulo Medical School from a lipid mixture composed of cholesteryl oleate, egg phosphatidylcholine (Lipoid, Ludwigshafen, Germany), triglycerides, cholesterol and 60 mg of derivatized MTX, according to the previously described method.^14^ Emulsification of the compounds was obtained by high-pressure homogenization using an Emulsiflex C5 homogenizer (Avestin Inc., Ottawa, ON, Canada) under apyrogenic conditions. The average particle size of LDE-MTX particles was 40‒60 nm, as measured by the dynamic light scattering method at a 90°angle, using the ZetaSizer Nano ZS90 equipment (Malvern Instruments, Malvern, UK). The LDE preparation was sterilized by passing through a 0.22 μm pore polycarbonate filter (Merck Millipore, Burlington, MA, USA) and was kept at 4 °C until it was used.

### LDE tissue uptake by the brain parenchyma

For the LDE tissue uptake study, the same LDE preparation was used, except that the lipids were labeled with [3H]-cholesteryl oleate ether (American Radiolabeled Chemicals, St Louis, MO, USA) and without MTX addition. Encephalon and liver tissues were collected from the animals of the Ischemic and of the Control groups 12 h after the injection of labeled LDE.

Tissue samples were kept in a cold saline solution before lipid extraction with chloroform/methanol (2:1 v/v).^26^ After extraction, the solvent was evaporated under N_2_ flow and resuspended with 500 mL of chloroform/methanol (2:1 v/v), and half the suspension was placed separately into vials with 5 mL of scintillation solution (Ultima Gold XR, Perkin Elmer). Levels of radioactivity were measured in the Liquid Scintillation Analyzer, 1200TR Tri-Carb, (Packard BioScience, Palo Alto, CA, USA).

### Tissue processing and perfusion

After 7 days post-injury, animals were deeply anesthetized (i.p) with a mixture of ketamine hydrochloride (72 mg/kg) and xylazine (9 mg/kg). After the absence of both corneal and paw withdraw reflexes, animals were perfused with 0.9 % saline solution followed by 4 % paraformaldehyde. Brains were removed and post-fixed for 24 h in the same fixative used in the perfusion and cryoprotected in gradients of a solution containing glycerol and sucrose. They were then embedded in Tissue Tek, frozen at −55 °C degrees in a cryostat chamber (Carl Zeiss, Oberkochen, Germany), and cut at 30 μm thickness. Coronal sections of 50 μm were also obtained for histopathological analysis using cresyl violet staining. Sections were directly collected onto gelatinized slides for better adherence and kept at room temperature for at least 24 h until being kept in a freezer at −20 °C.

### Histopathological analysis

The lesion area was assessed in 50 μm sections stained by cresyl violet (Sigma-Aldrich, Saint Louis, MO, USA). The sites of ET-1 injections were recognized by the presence of colanyl blue, pallor, inflammatory infiltrate and necrosis, as expected following ischemic damage.^23^

All stained sections were analyzed using light microscopy (Nikon Eclipse 50i, Nikon, Tokyo, Japan). High-resolution images from the more representative fields were obtained using a digital camera (Moticam 2500) attached to the light microscope.

### Immunohistochemistry

Previously established neuropathological parameters were measured to assess neuroprotection in different experimental models of acute brain^10^^,^^11^^,^^23^^,^^27^ and spinal cord injuries.^28^

To investigate effects of LDE-MTX on neuroinflammation and neuroprotection after cortical ischemia, the following antibodies were used: anti-NeuN (1:100, Chemicon, São Paulo, Brazil) as a marker for mature neurons; Anti-Iba1 (1:1000, Dako-Agilent, Santa Clara, CA, USA) as a specific microglial marker; anti-ED-1 (1:200, Bio-Rad, Hercules, CA, USA) as a marker of activated macrophages/microglia; anti-Glial Acid Fibrillary Protein (GFAP) (1:1000, Dako-Agilent), a classical astrocyte marker.

In brief, mounted sections on gelatinized slides were removed from the freezer, dried in an oven at 37 °C for 30 min., washed in Phosphate-Buffered Saline (PBS) with constant agitation for 5 min., and then immersed in borate buffer (0.2 M, pH 9.0) at 65 °C for 20 min.. Sections were then cooled in the same solution for the same period at room temperature. Sections were then washed three times in PBS for 5 min. and immersed in methanol containing hydrogen peroxide (1/100 mL of methanol).^24^

After antigen retrieval, sections were washed 3 times in PBS/Tween (5 min. each) and incubated in 10 % normal goat serum (anti-Iba1 and anti-GFAP) or normal horse serum (anti-ED1 and anti-NeuN) for 1 hour. After this period, they were incubated in primary antibody diluted in 10 % normal serum overnight using the previously mentioned dilution. On the next day, sections were washed again (3 times in PBS/Tween, 5 min. each) and incubated in biotinylated goat anti-rabbit or biotinylated horse anti-mouse (1:100) diluted in PBS for 2 h (1:200, Vector, Burlingame, CA, USA).

One hour before incubation in the secondary antibody, ABC solution (avidin-biotin-peroxidase-ABC Elite kit, Vector laboratories) was prepared and allowed to rest at least for 30 min. with no agitation. After further washing, sections were incubated in ABC solution diluted in PBS for 2 h. They were then washed four times and stained using diaminobenzidine (DAB, Sigma-Aldrich). Subsequently, sections were washed three times in 0.1 M PBS, dehydrated in alcohol gradients, cleared in xylene and coversliped using Entellan (Merck Millipore). All sections for the different antibodies were immunolabeled on the same day.

### Qualitative analysis

All sections stained with the different histological methods were observed using a light microscopy. High resolution images from the most representative fields were obtained using a digital camera (Moticam 2500, Motic Instruments, Canada) attached to the light microscope.

### Quantitative analysis

Numbers of NeuN+ cells and round activated macrophages/microglia (Iba1+ cells) cells were counted using a square 0.25 mm-wide grid (objective 40 ×) in the eyepiece of a microscope for all experimental groups. This grid corresponds to an area of 0.0625 mm^2^. It analyzed five animals per survival time, three sections per animal, 3‒4 fields per section. For NeuN+ cells, four counting fields in both the lateral and medial part of the periinfarct area, as the ischemic core is almost devoid of neuronal bodies were analyzed.

In the quantitative analysis of Iba1+ cells, three non-overlapping fields were counted in the ischemic core (3 sections per animal), where the highest number of rounded phagocytes is found, following the protocol previously reported by our group.^10^ This procedure aimed to quantitatively assess the effect of LDE-MTX on the maximal morphological level of microglial activation.^8^^,^^10^ Activated microglia becomes rounded phagocytes after ischemia, trauma, and other neural disorders,^8^ although a mix of microglia-derived and blood-borne macrophages are also present.^29^ Rounded Iba1+ cells represent a higher degree of morphological activation^8^^,^^24^ and correspond to the same population of activated microglia/macrophages labeled by anti-ED1+ cells.

For both Iba1 and NeuN analysis, adjacent sections to the lesion core were used. The lesion core was defined in 50 µm sections stained with cresyl violet. Investigators were blind to the experimental groups, and slide identifications were covered during counting.

### Statistical analysis

Shapiro–Wilk normality test was applied to the data. Descriptive statistic was performed for all counts. Averages, standard deviations, and standard errors were calculated. Comparisons between groups were assessed by unpaired Student´s *t*-test. The statistical significance level was accepted at *p* < 0.01. For data obtained from LDE tissue uptake, the following non-parametric tests were applied: Mann–Whitney and Kruskal-Willis tests. The statistical significance level was accepted at *p* < 0.01.

Data were tested for normality, and comparisons between groups were performed using an unpaired Student's *t*-test. For data on LDE tissue uptake and LDE–MTX treatment, the Mann–Whitney *U* test was applied. Data are expressed as mean ± SD (standard deviation) and in all analyses, *p* < 0.05 was considered statistically significant. All statistical analyses were performed using the GraphPad Prism 7.0 software (GraphPad Software, La Jolla, CA, USA).

## Results

### LDE tissue uptake

Table 1 shows the uptake of LDE labeled with 3H-cholesteryl oleate ether by the encephalon and the liver. As the liver is the major uptake site for LDE, the values of tissue uptake were expressed as a percentage (%) of the injected radioactivity per gram of encephalon tissue relative to the liver tissue uptake. LDE uptake by the encephalon of the ischemic group (3.4 ± 4.5) was fivefold higher than that of the encephalon of the control group (0.7 ± 0.6) (*p* = 0.0003).

**Table 1:** The uptake of LDE labeled with 3H-cholesteryl oleate ether by the encephalon and the liver.

	Animal number	Brain (CPM)	Liver (CPM)	Brain % uptake^a^^,^^b^
**Control**	1	35	9450	0.4
	2	36	9926	0.4
	3	74	9789	0.8
	4	35	9847	0.4
	5	61	9807	0.6
	6	15	8304	0.2
	7	80	9864	0.8
	8	77	9767	0.8
	9	33	9957	0.3
	10	14	9469	0.1
	11	41	1779	2.3
	12	16	934	1.7
	13	19	3035	0.6
	14	10	1004	1.0
	15	12	9574	0.1
	16	18	9247	0.2
	17	38	8681	0.4
	18	42	2238	1.9
	**mean ± SD**	**36 ± 30**	**7371 ± 3607**	**0.7 ± 0.6**
**Ischemic**	19	13	550	2.4
	20	31	812	3.8
	21	7	652	1.1
	22	17	719	2.4
	23	55	2423	2.3
	24	18	1578	1.1
	25	20	2159	0.9
	26	27	668	4.0
	27	19	827	2.3
	28	74	442	16.7
	29	20	2540	0.8
	**mean ± SD**	**27 ± 20**	**1216 ± 803**	**3.4 ± 4.5**

CPM, Counts Per Minute.

a The brain uptake is expressed as % of the liver uptake.

b p = 0.0003 (Mann-Whitney *U test*).

### Effect of LDE-MTX on inflammatory infiltrate

**Fig. 1:**
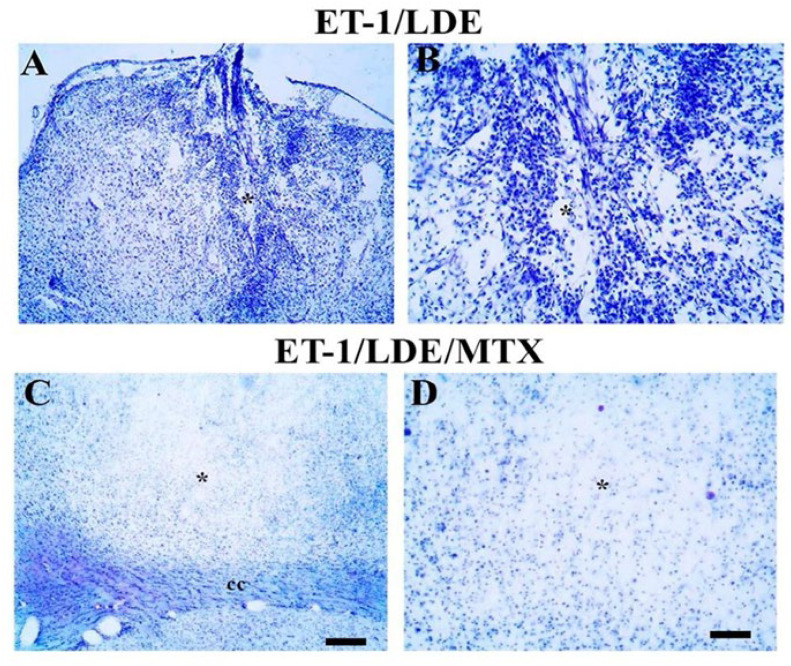
**Effects of LDE-alone (A‒B) and LDE-MTX (C‒D) treatment on the inflammatory infiltrate after frontal ischemia induction by Endothelin-1 (ET-1) in rats.** Representative photomicrographs of ischemic areas of stroke showing inflammatory cells stained by colanyl-blue stain. Ischemic animals were treated with LDE-alone (A, B) or treated with LDE-MTX (C, D). Asterisks are in the lesion epicenter. Scale bars represents: A and C (200 µm); B and D (80 µm).

Results showed that the ischemic animals treated with LDE only (i.e., LDE-alone group) presented classic focal ischemic damage, with tissue pallor and intense inflammatory response characterized by the presence of mononuclear cells in the ischemic core (Fig. 1A‒B). LDE-MTX group presented a great abolishment of inflammatory infiltrate in the ischemic core (Fig. 1C‒D). The two groups showed similar areas of primary infarct, which suggests that LDE-MTX treatment was not effective in reducing the primary infarct damage.

### Effect of LDE–MTX on microglial activation

**Fig. 2:**
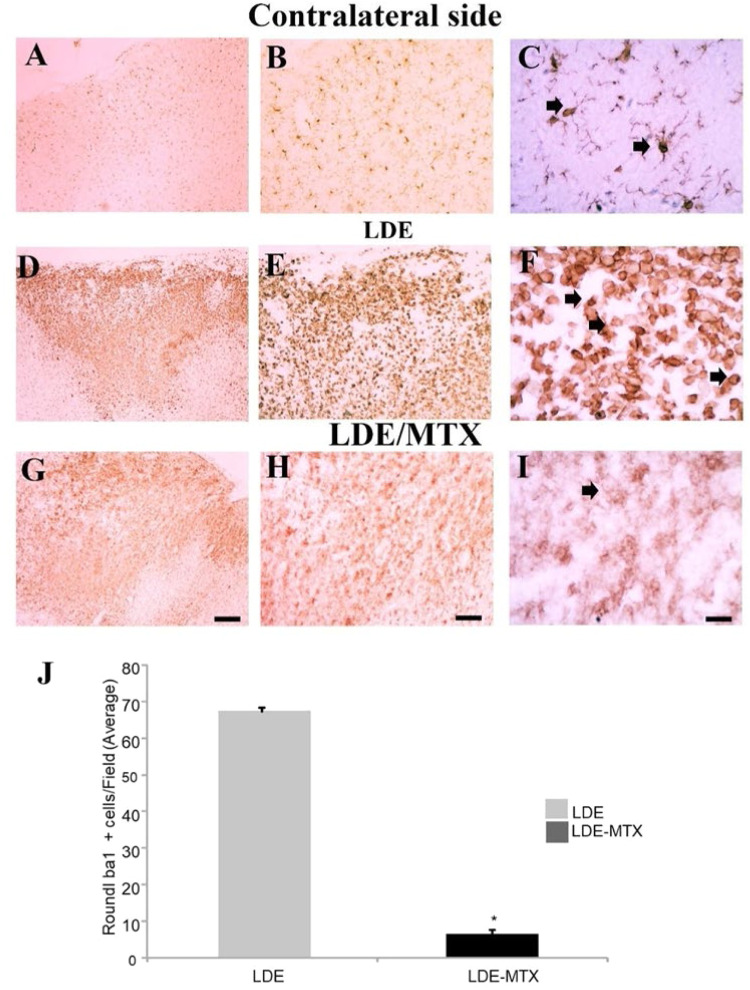
**Effects of LDE-alone (D-F) and LDE-MTX (G-H) treatment on microglial activation after frontal ischemia induction by ET-1 in rats.** Representative photomicrographs of ischemic and non-ischemic areas stained for ionized calcium-binding adapter molecule 1 (Iba1) in following groups: (A‒C) control animals injected with sterile saline showing inactivated microglia; (D‒F) ischemic animals treated with LDE-alone showing presence of microglial activation; and (G‒I): ischemic animals treated with LDE-MTX showing markedly reducing of this process. Arrows point to Iba1+ round macrophages (C, F, I). Scale bars: A, D, G: 200 µm; B, E, H: 80 µm; C, F, I: 20 µm. (J) Quantitative analysis of Iba1+ round cells showing a reduction of stained cell number in LDE-MTX compared to LDE group. * *p* < 0.01.

Ramified microglial were predominant in the contralateral side (Fig. 2A‒C). There was intense microglial activation in ischemic animals treated with LDE only (i.e., LDE-alone group) (Fig. 2D‒F). In these animals, there was a predominance of round microglia with phagocytic morphology at the ischemic core (Fig. 2E‒F). In the periphery of the lesion, microglia with amoeboid morphology could be observed (data not shown).

LDE-MTX treatment considerably reduced microglial activation (Fig. 2G‒I). There was a great decrease in the amount of round microglia at the lesion center as well as amoeboid microglia around the ischemic core (Fig. 2H‒J, indicating that LDE-MTX is a potent microglial inhibitor.

There was a great decrease in the number of Iba1+ round cells in the LDE-MTX group (6.55 ± 1.28) compared to the LDE-alone group (67.17±4.19) (Fig. 2J, *p* < 0.01). This data corresponds to a 90.25 % reduction in the number of highly activated microglia.

### Effect of LDE–MTX on astrocytosis

**Fig. 3:**
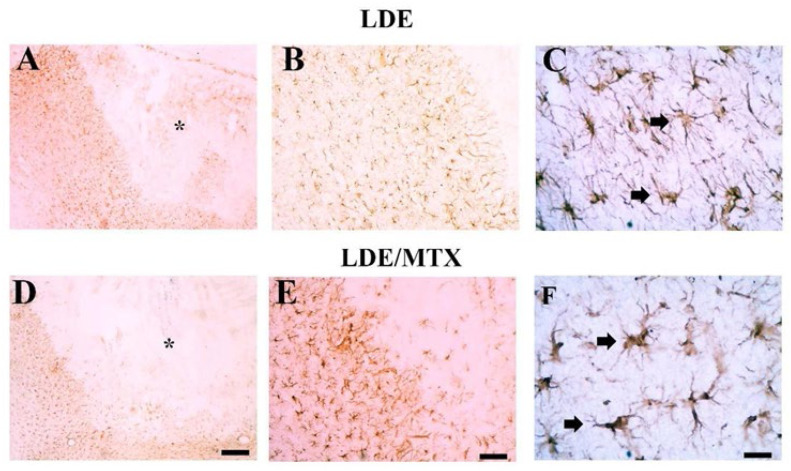
**Effects of LDE-alone (A‒C) and LDE-MTX (D‒F) treatment on astrocytosis process after frontal ischemia induction by ET-1 in rats.** Representative photomicrographs of ischemic areas of stroke stained for Glial Acid Fibrillary Protein (GFAP) in animals treated with LDE-alone (A‒C) or treated with LDE-MTX (D‒F). A similar pattern of astrocytosis was found in animals treated with LDE-alone and with LDE-MTX, indicating that treatment has no effect on astrocytosis. Arrows point to GFAP+ cells. Asterisks are in the lesion epicenter. Scale bars represent: A, D: 200 µm; B, E: 80 µm; C, F: 20 µm.

It was evaluated the effect of LDE-MTX treatment on astrocytosis in the proposed experimental model. Ischemic animals treated with LDE only (i.e., LDE-alone group) showed absence of astrocytes at the ischemic core (Fig. 3A) and intense astrocytosis at the periphery of the ischemic lesion (Fig. 3B‒C). These activated astrocytes were present in both gray and white matter, including corpus callosum (Fig. 3B‒C). A similar pattern of astrocytosis was found in animals treated with the LDE-MTX (Fig. 3D‒F), indicating that treatment has no effect on astrocytosis. Considering the quite similar patterns of astrocyte reactivity, the authors did not quantify the number of astrocytes.

### Effect of LDE–MTX on neuronal preservation

**Fig. 4:**
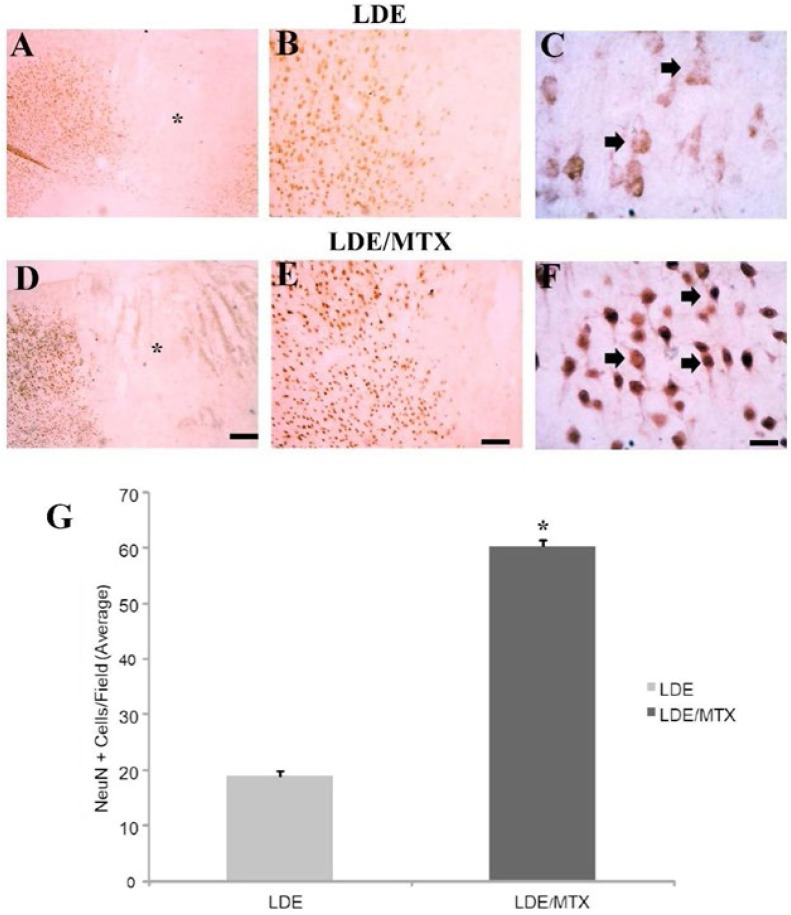
**Effects of LDE-alone (A‒C) and LDE-MTX (D‒F) treatment on neuronal preservation after frontal ischaemia induction by ET-1 in rats.** Representative photomicrographs of ischemic areas of stroke stained for NeuN, a neuronal marker, in animals treated with LDE-alone (A‒C) or treated with LDE-MTX (D‒F). There was no difference between areas of ischemic injury comparing LDE-alone group (A) and LDE-MTX (D). There was a considerable preservation of NeuN+ cell bodies in the periinfarct region in LDE-MTX group (E‒F) compared to LDE-alone group (B‒C). Arrows point to NeuN+ cell bodies. Asterisks are in the lesion epicenter. Scale bars represents: A, D: 200 µm; B, E: 80 µm; C, F: 20 µm. (G) Quantitative analysis of NeuN+ cell bodies showing a markedly increasing of neurons cells in the periinfarcted region of animals treated with LDE-MTX compared to those treated with LDE-alone. * *p* < 0.01.

It also explored the effect of LDE-MTX treatment on neuronal preservation after cortical ischemia. ET-1 microinjections into the motor cortex of rats induced conspicuous ischemic damage with conspicuous neuronal loss at the ischemic core (Fig. 4). There was no difference between areas of primary ischemic injury comparing LDE-MTX and LDE-alone groups (Fig. 4A and D, respectively). However, there was a considerable preservation of NeuN+ cell bodies in the periinfarct region in LDE-MTX (Fig. 4E‒F) compared to the LDE-alone group (Fig. 4B‒C).

In this respect, the quantification of NeuN+ cell body in the periinfarct region (Fig. 4G) revealed that the LDE-MTX group presented higher numbers of neuronal bodies per field (60.28 ± 1.37) compared to the LDE-alone group (18.86 ± 0.88) (*p* < 0.01). This data corresponds to a 319 % increase in neuronal preservation, suggesting a considerable neuroprotective effect of LDE-MTX.

## Discussion

Crossing the blood-brain barrier constitutes a chief challenge to nanoparticle systems aimed to deliver drugs to the encephalon. In this study, the feasibility of using LDE as a drug carrier for injured brain tissues was shown by the finding that the nanoparticles are taken up severalfold more by the encephalon of ET-1 treated animals than by the encephalon of the controls. This finding most likely suggests that disruption of the blood-brain barrier by ET-1 facilitated the entry of the LDE into the injured brain tissue. Likewise, the responses to LDE-MTX treatment observed here strongly suggest that a considerable amount of MTX was delivered to the target tissue to produce pharmacological effects. Recently, in patients with benign and malignant brain tumors submitted to intracranial surgery, the authors showed that LDE had the ability to concentrate in neoplastic and normal encephalic tissues after intravenous injection.^30^

Since it is well established that exacerbated microglial activation contributes to the damage secondary to focal ischemia, the effects of LDE-MTX treatment on microglial activation were explored in this study. In fact, the results unraveled LDE-MTX as a potent microglial inhibitor, rendering a tenfold decrease in the number of activated macrophages. More importantly, LDE-MTX treatment resulted in marked neuroprotection in the periinfarction region, supported by the preservation of threefold more NeuN+ cell bodies.

LDE-MTX treatment did not affect the primary infarct damage resulting from the constriction of the vessels by ET-1 microinjections. This was different from the effects of the treatment with this formulation on rats with myocardial infarction induced by ligation of the left coronary artery. In those experiments, reduction of the infarcted area indeed occurred within a 30-day observation period.^14^ To account for the differences in the results between the referred myocardial and the cerebral infarct rat models, it can be pointed out that cell death and necrosis in the brain ischemic core occur quickly, which renders this region scarcely amenable to neuroprotective agents to restore the damaged tissue. In animal studies, the treatment with other drugs known to act on ischemic strokes, such as indomethacin,^31^ PJ34, and minocycline^32^ was not effective in decreasing the infarction area. In contrast, previous studies showed that treatment with minocycline was able to considerably reduce the area of cortical^24^ and striatal infarction, using the experimental model of middle cerebral artery occlusion in adult ischemic rats. It is noteworthy to mention that in successful thrombolytic therapy performed in human patients, the size of the infarction remains unchanged, as shown by Magnetic Resonance Imaging (MRI).^33^

Thrombolytic therapies aim to rescue the threatened brain tissue, termed “tissue at risk” or the ischemic penumbra. However, this therapeutic approach is not beneficial for all patients of ischemic stroke and with time, the infarct core expands into the ischemic penumbra, and therapeutic opportunity is lost.^3^ Mismatch MRI shows that this expansion illustrates the considerable amount of salvageable neural tissue amenable to neuroprotection.^27^ The counterpart of this phenomenon in experimental models of stroke is illustrated by the progressive loss of NeuN+ cell bodies in the periinfarction area. Here, the authors showed considerable neuroprotection achieved by LDE-MTX in the periinfarction area.

The mechanisms whereby LDE-MTX might protect neurons in the peripherical zone of the infarct should be explored in future studies. Nonetheless, previous studies on the action of this formulation may shed some light on this issue. MTX is a folate analogue with antiproliferative, and immunosuppressive activity used in cancer treatment and in autoimmune inflammatory diseases.^28^ The anti-inflammatory effect of MTX seems to occur through multiple actions, including folic antagonism, inhibition of eicosanoids, metalloproteinases, adenosine accumulation, and blockage of lymphocyte proliferation with the corresponding production of Tumor Necrosis Factor-α (TNF-α), Interleukin (IL) −8 and IL-12, among others.^34^

The uptake of MTX by cells through the folate receptor is deficient and is considered a major drawback in MTX pharmacology. In the study by Moura et al.,^21^ it was shown that the incorporation of MTX to LDE resulted in a ninetyfold increase in the cellular uptake of the drug, consequent to the substitution of the LDL receptor endocytic pathway for the folate uptake mechanism. By comparing the treatment with LDE-MTX with that of conventional MTX, as administered in a rabbit model of rheumatoid arthritis, it was demonstrated that LDE-MTX was markedly superior in diminishing the number of inflammatory cells in the synovial fluid and in promoting the reduction of the inflammation in the synovial tissue, probably due to the massive entry of MTX into the cells facilitated by LDE as vehicle.^12^

In rats with myocardial infarction, treatment with LDE-MTX produced marked beneficial effects on the non-infarcted area of the heart, whereas the commercial MTX action was practically null. The remarkable improvement in post-infarction condition was probably related to the ability of LDE-MTX to efficiently improve the bioavailability or release of adenosine by increasing angiogenesis, thereby decreasing hypoxia.^14^ The lack of effect of commercial MTX on myocardial infarction induced in rats^14^ and the fact that commercial MTX bears high toxicity to human subjects justifies the non-inclusion of a group of rats treated with the commercial formulation in our current study protocol. Recently, the good tolerability and lack of clinical and laboratory toxicity of LDE-MTX were documented in women with endometriosis.^35^

In this study, it was remarkable that LDE-MTX treatment reduced the microglial activation at a degree not previously afforded by any anti-inflammatory drug tested for central nervous system diseases. Although microglial cells have beneficial and neuroprotective effects after ischemia,^36^ their detrimental effects are well described after uncontrolled activation in both acute^37^ and chronic neural disorders.^29^ It is likely that intense microglial modulation provided by LDE-MTX is fundamental for the considerable neuroprotection reported here. In this regard, minocycline affected microglial activation; however, only the combined therapy of minocycline with bone marrow mononuclear cells produced effects that can be considered comparable to our current results.^24^

Although LDE-MTX conspicuously modulates microglial reactivity, there was no observable effect of this formulation on astrocytosis. This suggests that the neuroprotective effects afforded by LDE-MTX are not related to the modulation of astroglia function. In classical studies.^25^^,^^29^ and in experimental models of hemorrhagic stroke minocycline also showed any effect on astrocytosis. However, a recent study suggests that minocycline increases astrocyte activation following photothrombosis in the sensory system of adult rats contributing to neuroprotection.^33^

The relatively small number of animals used in each experimental group (treated with LDE-MTX and controls) might perhaps have narrowed the statistical power of the results and may be pointed out as a limitation of the study. The choice of a seven-day observation period is because this is the time in which the lesions are maximum in animal models of encephalic ischemia.^31^ In future studies, it should be interesting to extend the observation period to seize the sustainability of the treatment outcomes.

## Conclusions

Our findings suggest that LDE-MTX is a potent anti-neuroinflammatory formulation, achieving a pronounced reduction of microglial activation. Moreover, LDE-MTX treatment had neuroprotective action, shown by the marked neuronal preservation in the penumbra area, without affecting astrocytosis. These results from the rat model of ET-1-induced ischemic stroke justify further pre-clinical studies of LDE-MTX. Given the scarcity of effective treatments for ischemic stroke today, this nanomedicine approach offers a promising novel therapeutic tool.

## Consent for publication

Not applicable.

## Authors’ contributions

Study concept and design: Edmundo L. R. Pereira, Michelle N. C. Dias, Ijair R. dos Santos, Carolina Ramos dos Santos, Moisés Hamoy, Danielle Cristine A. Feio. Acquisition of data: Michelle N. C. Dias, Ijair R. dos Santos, Carolina Ramos dos Santos, Moisés Hamoy, Danielle Cristine A. Feio, Aleksandra T. Morikawa Priscila O. Carvalho. Analysis and interpretation of data: Edmundo L. R. Pereira, Michelle N. C. Dias, Ijair R. dos Santos, Carolina Ramos dos Santos, Moisés Hamoy, Danielle Cristine A. Feio. Conceptualization, Methodology, Validation, Formal analysis, Writing-original draft, Writing-review & editing: Raul C. Maranhão, Priscila O. Carvalho, Jaqueline M. Bazioli, Aleksandra T. Morikawa, Walace Gomes-Leal. All authors participated in the critical review and approval of the final draft submitted.

## Funding

This study was supported by the Brazilian National Council for Scientific and Technological Development (CNPq-Brazil) and 10.13039/501100001807Fundação de Amparo à Pesquisa do Estado de São Paulo (2020/16215-0).

## Conflicts of interest

The authors declare no conflicts of interest.
